# Reshaping the immunosuppressive glioma microenvironment: mechanisms, biomarkers, and emerging immunotherapies

**DOI:** 10.3389/fimmu.2026.1819951

**Published:** 2026-04-16

**Authors:** Zheng Hu, Jun Luo, Jianyun Lou, Juntao Deng, Zitao Gong, Jinming Chen

**Affiliations:** 1 The First Affiliated Hospital of Gannan Medical University, Ganzhou, Jiangxi, China; 2 Department of Neurosurgery, The First Affiliated Hospital of Gannan Medical University, Ganzhou, Jiangxi, China; 3 Gannan Medical University, Ganzhou, Jiangxi, China

**Keywords:** biomarkers, combination therapy, glioma, immune suppression, immunotherapy, myeloid cells, patient stratification, tumor immune microenvironment

## Abstract

Gliomas encompass biologically distinct subtypes with different clinical behaviors and immune microenvironments. IDH-wildtype glioblastoma (GBM), the most aggressive subtype, has a median survival of 14–18 months, while IDH-mutant gliomas (including grade 2–4 astrocytomas and oligodendrogliomas) have significantly better prognoses (median OS 3–8 years; Cancer Genome Atlas, NEJM 2015). Throughout this review, we specify the glioma subtype when discussing each mechanism and therapeutic strategy. Although immunotherapy has achieved major breakthroughs in multiple solid tumors, its clinical efficacy in glioma remains consistently limited. Accumulating evidence indicates that a profoundly immunosuppressive tumor immune microenvironment is the principal barrier to successful immunotherapy in glioma. Therefore, systematically reshaping this immunosuppressive state has become a central research priority. This narrative review summarizes the establishment and regulatory mechanisms of the immunosuppressive microenvironment in glioma, outlining global immune characteristics and key cellular and molecular foundations. We focus on the myeloid-cell network dominated by tumor-associated macrophages (TAMs) and microglia, T-cell dysfunction, and the pivotal roles of immunosuppressive signaling pathways and metabolic reprogramming in sustaining immune suppression. In addition, we comprehensively review candidate biomarkers associated with immunotherapy response, including multidimensional indicators at molecular, cellular, and spatial levels, and emphasize their potential value for patient stratification and treatment decision-making. Building on this framework, we further analyze recent progress in emerging immunotherapeutic strategies for glioma—such as immune checkpoint inhibitors (ICIs), tumor vaccines, cellular immunotherapies, and oncolytic viruses—together with the major causes underlying their constrained efficacy. We reinterpret clinical outcomes through the lens of the immune microenvironment. By integrating available evidence, this review highlights the importance of combination regimens aimed at microenvironmental remodeling to overcome immune tolerance, and discusses key challenges and future directions. Overall, we propose that remodeling the immunosuppressive microenvironment—rather than merely enhancing immune activation—is a prerequisite for meaningful breakthroughs in glioma immunotherapy. A deeper understanding and more precise intervention of the immune microenvironment may yield clinically substantive benefits for patients with glioma.

## 1 Introduction

Gliomas are the most common primary malignant tumors of the CNS. High-grade gliomas, particularly GBM, are highly invasive and associated with extremely poor clinical outcomes (1). Despite continuous optimization of neurosurgical techniques, radiotherapy protocols, and chemotherapy regimens represented by temozolomide (TMZ), overall survival gains remain limited. Median survival is typically 14–18 months, and the 5-year survival rate is <10%, underscoring the urgent need for new therapeutic paradigms (2).

Immunotherapy, which has delivered transformative advances across multiple solid tumors, has raised hopes for glioma treatment. However, in stark contrast to tumors such as melanoma and non-small cell lung cancer, the overall efficacy of ICIs, tumor vaccines, and cellular immunotherapies in glioma has been largely disappointing (3). Numerous clinical trials suggest that single-agent immunotherapy rarely induces durable responses in glioma. This does not necessarily indicate intrinsic inefficacy of the modalities; rather, glioma exhibits distinct immunologic features, including the brain-specific immune context, a deeply myeloid-dominated immunosuppressive tumor microenvironment (TME), and tumor-intrinsic immune-evasion programs (4, 5). These characteristics distinguish glioma from other solid tumors.

Increasing evidence indicates that a profoundly immunosuppressive tumor immune microenvironment is the key bottleneck limiting immunotherapy in glioma. This microenvironment is characterized not only by insufficient infiltration of effector T cells but also by an immunosuppressive network dominated by TAMs and microglia, together with persistent activation of multiple immune checkpoint molecules, immunosuppressive cytokines, and metabolic dysfunction (6, 7). This complex and dynamic immunosuppressive state has led to glioma being widely regarded as a prototypical “immune-cold tumor” (8).

In this context, strategies that solely amplify immune activation are unlikely to meet clinical needs. A growing consensus suggests that achieving meaningful progress in glioma immunotherapy requires systematic, multi-pathway remodeling of the immunosuppressive microenvironment, rather than targeting a single checkpoint or a single cell type (9, 10). Current efforts include targeting immunosuppressive cells, fine-tuning immunometabolism, optimizing the timing of immunotherapy (e.g., neoadjuvant settings), and designing multimodal combination regimens to create more feasible routes toward clinical breakthroughs (11, 12).

Accordingly, this review centers on “remodeling the immunosuppressive microenvironment.” In a narrative format, we synthesize recent advances from basic and clinical studies, systematically delineate key immunologic mechanisms in the glioma immune microenvironment, summarize candidate biomarkers associated with immunotherapy response, and discuss emerging immunotherapies and combination strategies aimed at overcoming immune suppression. We further argue that it is necessary to integrate mechanistic insights, biomarkers, and clinical evidence into a decision framework built around “immune microenvironment subtype—combination strategy—dynamic monitoring indicators,” thereby providing an actionable theoretical basis for precision immunotherapy.

Recent comprehensive reviews have summarized the complex cellular interactions within the glioma microenvironment, providing an integrative overview of immune niches, cellular heterogeneity, and therapeutic implications (6, 9, 13).

## 2 Literature search and inclusion criteria

This review is a thematic narrative review and did not follow the Preferred Reporting Items for Systematic Reviews and Meta-Analyses (PRISMA) workflow to conduct a systematic review or meta-analysis. Studies eligible for inclusion comprised basic mechanistic research, translational studies, and clinical trials related to immunotherapy (including information from clinical trial registries). We primarily included English-language articles published in peer-reviewed journals. To minimize omissions, we performed reference tracking for key studies and conducted supplementary searches on . The literature search was performed mainly in PubMed, Web of Science, Embase, and , with a primary focus on articles published between January 2014 and January 2025. Foundational studies published before 2014 were also included when necessary to provide essential mechanistic context. Search terms included, but were not limited to: “glioma” OR “glioblastoma”; “tumor microenvironment” OR “immune microenvironment”; “immunotherapy” OR “immune checkpoint inhibitor” OR “vaccine” OR “CAR-T” OR “oncolytic virus”; “biomarker” OR “immune suppression”.

The inclusion criteria were as follows: (1) studies focusing on mechanisms of the glioma immune microenvironment; (2) studies reporting immunotherapy-related clinical trials or translational research; (3) we primarily included English-language articles published in peer-reviewed journals from the past 11 years (January 2014 to January 2025).

The exclusion criteria were: non-glioma studies; studies that were purely methodological descriptions lacking immune-related content; and duplicate records and/or overlapping reports.

## 3 Global features of the glioma immune microenvironment

As highlighted in recent integrative reviews, the glioma immune microenvironment is characterized by distinct niches that shape immune cell composition and function (13, 14).

### 3.1 Glioma as a prototypical “immune-cold tumor”

Overall, glioma is widely recognized as a prototypical “immune-cold tumor.” Compared with immunotherapy-responsive solid tumors, glioma tissues typically show markedly lower infiltration of effector immune cells—especially cluster of differentiation 8–positive (CD8^+^) cytotoxic T lymphocytes—whereas immunosuppressive cells and signaling pathways remain chronically activated (6, 15). This “low immune reactivity” is evident not only within the tumor but also in systemic immune dysfunction in the peripheral compartment.

The low immunogenicity of glioma is partly attributable to a relatively low tumor mutation burden (TMB) and limited neoantigen generation, rendering tumor cells less readily recognized as “non-self” (16). In addition, defects in antigen processing and presentation further impede effective immune recognition and activation. Together, these factors shape an intrinsic baseline of poor sensitivity to immunotherapy.

### 3.2 Immune particularities of the CNS

Glioma arises in the CNS, an organ environment with unique immunologic features. Historically, the brain was considered “immune-privileged.” Although this view has been revised, the CNS still differs substantially in immune-cell entry, antigen presentation, and inflammatory regulation (17). The blood–brain barrier (BBB) physiologically restricts peripheral immune cells and macromolecules from entering brain tissue; while partially disrupted in tumors, residual BBB function can still affect delivery and efficacy of immunotherapies (18).

Moreover, microglia—the resident innate immune cells of the brain—play essential roles in maintaining neural homeostasis. In the glioma microenvironment, however, microglia are often “re-educated” by tumor-derived signals toward phenotypes that support tumor growth and immune suppression (19, 20). These tissue-specific immune regulatory mechanisms confer immune-microenvironment features that differ from those of peripheral solid tumors (21).

### 3.3 Dynamic co-shaping between tumor and immune microenvironment

Importantly, the glioma immune microenvironment is not static but evolves dynamically (22). Tumor cells continuously reshape the local immune landscape by secreting cytokines, chemokines, and metabolic products; conversely, immune-cell changes feedback to influence tumor invasiveness, growth kinetics, and therapeutic response (23, 24).

As tumors progress and treatments are applied, the immune microenvironment can change substantially. Surgery, radiotherapy, and chemotherapy not only act on tumor cells but also influence immune-cell recruitment and function, sometimes even exacerbating immune suppression (22, 25). Understanding these tumor–therapy–immune interactions is critical for rational design of immunotherapy and combination strategies.

Collectively, the glioma immune microenvironment is characterized by low immunogenicity, strong immunosuppression, pronounced tissue specificity, and dynamic evolution. These features provide essential context for dissecting cellular and molecular mechanisms of immune suppression and for understanding repeated failures of immunotherapy in glioma.

### 3.4 Additional clinical determinants of immunosuppression

Beyond the local tumor microenvironment, several clinical and systemic factors contribute to immunotherapy failure in glioma. First, intratumoral heterogeneity and clonal evolution drive antigen loss and immune escape, particularly after targeted therapies such as CAR−T cells or peptide vaccines (26). Second, systemic immunosuppression is evident in GBM patients, including expansion of circulating MDSCs, impaired T−cell function, and lymphopenia induced by standard−of−care temozolomide (27). Third, the widespread use of corticosteroids (e.g., dexamethasone) to manage cerebral edema potently suppresses T−cell activation and survival (28). Fourth, radiotherapy and chemotherapy not only deplete tumor cells but also cause prolonged lymphopenia, which may negate the benefits of subsequent immunotherapy (29). These factors must be considered when interpreting clinical trial results and designing future combination regimens (6).

## 4 Key cellular and molecular mechanisms of the immunosuppressive glioma microenvironment

### 4.1 A myeloid-dominated immunosuppressive network

The heterogeneity of glioma−associated myeloid cells has been extensively characterized by single−cell studies, as summarized in recent reviews.

In glioma, myeloid cells constitute the most abundant and functionally influential immune population. Tumor-associated macrophages (TAMs) and microglia account for 30-50% of total tumor cells in IDH-wildtype GBM (range 25-65% across studies), with significant variation by molecular subtype. Mesenchymal GBM shows the highest TAM infiltration (median 45%, IQR 38-52%), while proneural GBM has relatively lower TAM abundance (median 28%, IQR 22-35%). In contrast, IDH-mutant gliomas exhibit significantly lower TAM infiltration (median 18%, IQR 12-25%) (30–32). Similar to many peripheral solid tumors, glioma-associated TAMs frequently display immunosuppressive phenotypes that promote tumor growth and blunt effector immunity (21).

Glioma cells recruit peripheral monocytes through secretion of colony-stimulating factor 1 (CSF-1) (33). In a genetically engineered mouse model of GBM, CSF-1R inhibition with PLX3397 reduced tumor-associated macrophages (TAMs) by >60% (from 45% to 18% of total cells, p<0.001) and prolonged median survival from 28 to 42 days (p<0.01). However, clinical translation of this strategy has been challenging. A phase II trial of PLX3397 in recurrent GBM (NCT01349036, n=37) showed acceptable safety but limited efficacy (6-month progression-free survival [PFS] 8.6%, median overall survival [OS] 7.2 months) (34), highlighting the redundancy of myeloid recruitment pathways. Meanwhile, resident microglia undergo phenotype switching in response to tumor cues, together forming a complex immunosuppressive network (35). These cells secrete immunosuppressive cytokines such as interleukin 10 (IL-10) and transforming growth factor beta (TGF-β), and express metabolic enzymes including arginase 1 (ARG1) and indoleamine 2,3-dioxygenase (IDO), thereby directly suppressing T-cell proliferation and effector function (36).

In addition to TAMs, TANs have emerged as important modulators of the glioma microenvironment (37, 38). Their abundance correlates with higher tumor grade and poorer prognosis. Similar to macrophages, neutrophils exhibit functional plasticity, with N1 (anti−tumor) and N2 (pro−tumor) polarization states (39). In glioma, TANs predominantly acquire an N2−like phenotype, promoting angiogenesis and immunosuppression (40). Crosstalk between TANs and TAMs further amplifies the immunosuppressive network (41). Targeting TANs represents an underexplored therapeutic avenue.

Notably, the conventional M1/M2 dichotomy is insufficient to capture the profound heterogeneity of glioma-associated myeloid cells (42). Single-cell sequencing studies show that TAMs exhibit continuum-like variation in spatial distribution, transcriptional programs, and functional states (43). This heterogeneity increases the complexity of therapeutic targeting and suggests that interventions against a single target are often inadequate to reverse immune suppression.

### 4.2 T-cell exhaustion and adaptive immune dysfunction

Although glioma is “immune-cold,” some T lymphocytes can still be detected within tumor tissues. However, these T cells are frequently dysfunctional or exhausted, limiting effective antitumor activity (44). Chronic antigen exposure and immunosuppressive signals drive high expression of multiple inhibitory receptors, including programmed cell death protein 1 (PD-1), T-cell immunoglobulin and mucin-domain containing-3 (TIM-3), lymphocyte activation gene 3 (LAG-3), and T-cell immunoreceptor with Ig and ITIM domains (TIGIT), accompanied by reduced cytotoxic molecules and impaired cytokine production (45).

This widespread T-cell exhaustion provides a key explanation for the limited efficacy of ICIs in glioma. Unlike other tumors, glioma typically exhibits both low T-cell abundance and multi-layered functional impairment, making single-checkpoint blockade insufficient to restore full effector activity (46, 47). Additionally, close crosstalk between immunosuppressive myeloid cells and exhausted T cells further aggravates failure of adaptive immunity (48).

### 4.3 Immunosuppressive signaling and metabolic reprogramming

Beyond cellular regulation, the glioma microenvironment is profoundly shaped by immunosuppressive signaling pathways and metabolic programs (49). Tumor and immune cells engage in metabolic reprogramming that creates a hostile local milieu for immune-cell survival and function. For example, high tumor glycolysis leads to lactate accumulation and local acidification, suppressing T-cell and natural killer (NK) cell activity (50, 51).

Meanwhile, aberrant activation of tryptophan metabolism via enzymes such as IDO depletes essential nutrients and generates immunosuppressive metabolites, further weakening immune responses (31). Hypoxia-induced pathways not only promote tumor invasion but also enhance expression of immunosuppressive molecules (52). These metabolic and molecular changes synergize with altered cellular composition to maintain an immunosuppressive steady state (53).

**Figure 1:**
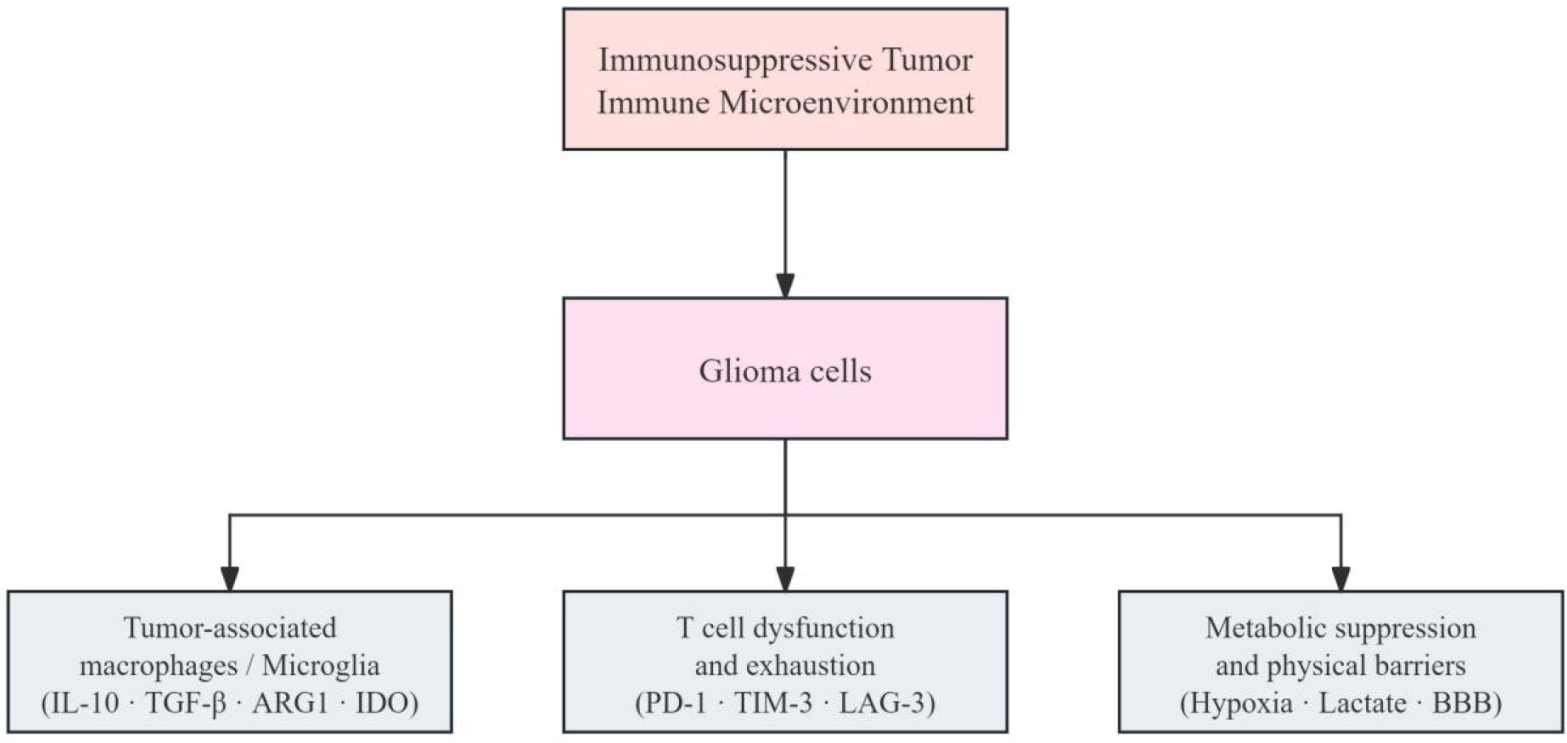
Composition of the immunosuppressive tumor immune microenvironment in glioma. Glioma harbors a highly immunosuppressive tumor immune microenvironment characterized by a myeloid-cell network dominated by TAMs and microglia, dysfunctional and exhausted T cells, and a suppressive milieu shaped by hypoxia, metabolic reprogramming, and the BBB. These factors act synergistically to constrain effective antitumor immunity and form a major basis for limited responses to immunotherapy.

In summary, the immunosuppressive glioma microenvironment is a multilayered system driven by myeloid dominance, impaired T-cell function, and convergent immunosuppressive signaling and metabolic reprogramming. These coordinated mechanisms explain persistent failures of immunotherapy and point to clear directions for “microenvironment remodeling” (**Figure 1**).

### 4.4 Controversies and unresolved questions

Despite progress, several key controversies remain. First, the dual role of TAMs is not fully understood. While TAMs are predominantly immunosuppressive in glioma, certain subsets may retain antitumor functions under specific conditions (35). Whether these cells can be reliably reprogrammed toward an immunostimulatory phenotype (e.g., via CD40 agonists or TLR ligands) without promoting inflammation−related toxicity remains an open question (54, 55).

Second, the failure of immune checkpoint inhibitors in glioma cannot be attributed solely to the local microenvironment. Tumor−intrinsic factors (e.g., PTEN loss, low neoantigen burden) (56), host−related factors (e.g., corticosteroid use, baseline lymphopenia, systemic MDSC expansion) (28, 57), and treatment−induced lymphopenia (e.g., from temozolomide) all contribute to resistance (27).

Third, the striking discordance between preclinical efficacy and clinical failure of CSF1R inhibitors highlights the redundancy of myeloid recruitment pathways and the emergence of compensatory suppressive populations (e.g., neutrophils, monocytic MDSCs) (34, 58).

These controversies underscore the need for combination strategies that target multiple nodes simultaneously and for trial designs that incorporate comprehensive biomarker profiling (6, 9).

## 5 Immune microenvironment–related biomarkers and clinical implications

With expanding efforts in glioma immunotherapy, identifying biomarkers that predict treatment response and reflect immune-microenvironment states has become a major research focus. Compared with other solid tumors, glioma still lacks widely validated immune-related biomarkers that can be applied robustly in clinical decision-making, which limits optimization and personalization of immunotherapeutic strategies. To facilitate multi-level interpretation of biomarker utility in patient stratification and treatment selection, we summarize candidate biomarkers at molecular, cellular, spatial, and dynamic levels (**Table 1**).

**Table 1:** A layered integrative framework of immune-related biomarkers in glioma.

Level	Representative biomarker	Biological significance	Potential clinical use	Clinical evidence level (with references)
Molecular	IDH mutation	Reflects tumor metabolic state, associated with better prognosis	Baseline patient stratification; inclusion criterion for trials	Validated prognostic marker, used in trials (Phase III) (59)
Molecular	MGMT methylation	Predicts response to temozolomide; associated with hypermutation	Chemotherapy guidance; exploratory for immunotherapy selection	Established predictive marker for TMZ, exploratory for immunotherapy (60)
Molecular	TMB	Low in most gliomas; reflects neoantigen burden	Limited utility; hypermutated tumors may benefit from ICI	Low in most gliomas, limited utility (56)
Molecular	PD-L1 expression	Immune checkpoint ligand; correlates with immune infiltration	Not recommended for patient selection in glioma	Inconsistent predictive value, not recommended for patient selection (61)
Cellular	TAMs/MDSCs	Major immunosuppressive population; correlates with poor prognosis	Assess need for myeloid-targeting therapies	Strong preclinical rationale, prospective validation ongoing (33)
Cellular	CD8^+^ T-cell infiltration	Effector T cells; indicates immune activation	Prognostic; predictive value requires functional assessment	Prognostic, predictive value limited without functional assessment (62)
Cellular	Regulatory T cells	Immunosuppressive T cells; promote immune tolerance	Potential target for depletion	Prognostic, but predictive value unclear (preclinical evidence)
Spatial	Spatial CD8/TAM ratio	Reflects immune exclusion and “cold tumor” phenotype	Guide local therapies; emerging biomarker	Emerging biomarker, being evaluated in correlative studies (43)
Dynamic	ctDNA dynamics	Real-time monitoring of tumor burden and immune changes	Monitor treatment response; guide adaptive therapy	Exploratory, being integrated into ongoing trials (63)

IDH, isocitrate dehydrogenase; MGMT, O^6^-methylguanine-DNA methyltransferase; TMZ, temozolomide; TMB, tumor mutational burden; ICI, immune checkpoint inhibitor; PD-L1, programmed death-ligand 1; TAMs, tumor-associated macrophages; MDSCs, myeloid-derived suppressor cells; CD8, cluster of differentiation 8; Tregs, regulatory T cells; ctDNA, circulating tumor DNA.

References cited in the “Clinical Evidence Level” column correspond to the reference list at the end of the manuscript.

Accordingly, we outline biomarkers across the four levels and emphasize multidimensional integration for patient stratification to inform immunotherapy selection and combination design.

### 5.1 Molecular-level immune-related biomarkers

Expression of immune checkpoint molecules is among the most extensively studied candidate predictive biomarkers. PD-L1 is detectable in a subset of gliomas, and its level correlates to some extent with tumor grade, immune infiltration, and prognosis (64). However, multiple clinical studies indicate that PD-L1 expression does not reliably predict benefit from ICIs in glioma, underscoring that a single molecular marker cannot capture the complexity of immune suppression (65).

TMB and neoantigen burden are key determinants of immunotherapy response in many cancers, yet glioma generally has low mutational burden; only small hypermutated subgroups may benefit (56). In addition, IDH mutation status is closely linked to immune-microenvironment features. IDH-mutant gliomas often show reduced immune infiltration and enhanced immune suppression, making IDH status a potentially useful stratification factor for immunotherapy (31). Molecular biomarkers are susceptible to regional sampling and therapy-induced shifts; recommended composite readout: interpret baseline IDH/TMB/PD-L1 together with myeloid burden and T-cell functional state, preferably anchored by a spatial metric (e.g., CD8/TAM ratio).

### 5.2 Cellular composition–related biomarkers

Immune-cell composition and distribution are important indicators of immune-microenvironment status. CD8^+^ T-cell infiltration correlates with prognosis and immunotherapy response in some studies, but predictive value is strongly influenced by functional state (46). CD8^+^ T-cell counts alone cannot distinguish effective effector populations from terminally exhausted populations and therefore are insufficient as standalone predictors; functional, clonal, and spatial parameters are required for comprehensive evaluation (66).

Myeloid biomarkers have attracted growing attention. TAM density, cellular origin (peripheral monocyte-derived vs resident microglia), and functional phenotype are considered crucial determinants of immunotherapy outcomes (35). High proportions of immunosuppressive TAMs/MDSCs are closely associated with resistance to ICIs and chimeric antigen receptor T-cell (CAR-T) therapy, providing biological and preclinical support for myeloid-targeted combination or sequential immunotherapy strategies (67, 68). Immune-cell quantification is confounded by spatial heterogeneity (core vs margin) and does not necessarily reflect functionality; recommended composite readout: CD8 abundance plus exhaustion signature, together with TAM/MDSC load and a spatial CD8/TAM ratio, supplemented by peripheral monitoring when available.

### 5.3 Emerging dynamic and spatial biomarkers enabled by new technologies

Single-cell sequencing, spatial transcriptomics, and multiplex immunofluorescence have substantially advanced understanding of glioma immune complexity. These approaches not only resolve immune-cell heterogeneity but also reveal spatial organization of distinct cell subsets within tumor architecture, enabling more accurate assessment of immunosuppressive network organization (69, 70).

Dynamic monitoring—such as pre- and post-treatment shifts in immune-cell composition and functional states—is also considered a promising direction for predicting immunotherapy response. Compared with static, single time-point measurements, longitudinal profiling of microenvironment evolution may better identify patients who truly benefit from immunotherapy (25, 71).

Overall, biomarker research in glioma remains exploratory. The key future priority is to integrate molecular, cellular, and spatial information to construct composite predictive models that reflect holistic immune-microenvironment states and guide precision immunotherapy and remodeling strategies (22). Existing evidence suggests that microenvironment features reflecting degree of immune suppression—such as myeloid infiltration patterns, T-cell functional phenotypes, and activation of metabolic pathways—may offer greater integrative value than single-gene expression indicators (6, 72). Multi-omics integration with spatial information to define immune-microenvironment phenotypes may become the foundation for patient stratification and combination design. Platform-specific biases (scRNA-seq, spatial transcriptomics, multiplex IF) and batch effects limit cross-cohort comparability; recommended composite readout: harmonize a minimal core set (spatial CD8/TAM ratio + exhaustion program + peripheral immune dynamics) for cross-validation.

### 5.4 Toward an integrative immune microenvironment biomarker model

Although the biomarkers above show investigational value, current evidence consistently indicates that a single biomarker is insufficient to represent the complex and profoundly immunosuppressive tumor immune microenvironment of glioma. Predictive performance is limited for PD-L1, TMB, and infiltration of individual immune subsets (6, 56), implying that immune evasion in glioma is driven by coordinated multi-level regulation rather than a single mechanism.

Thus, moving from single markers toward multidimensional integration is becoming a major direction in the field. Conceptually, integrating PD-L1, TMB, IDH status, TAM features, and spatial/dynamic information may define distinct immune-microenvironment subtypes (43). Such classification could help explain marked inter-patient variability in immunotherapy response and provide a biological basis for rational selection of immunotherapy and combination strategies. Accordingly, clinical immunotherapy should emphasize stratification–matching, rather than one-size-fits-all regimens. A clinically actionable composite panel may integrate baseline molecular features (e.g., IDH/TMB/PD-L1), spatial immune architecture (e.g., CD8/TAM ratio), T-cell dysfunction signatures, and dynamic peripheral readouts (e.g., cytokines/ctDNA) to guide combination selection and on-treatment adaptation. Recommended composite (pragmatic): baseline IDH/TMB/PD-L1 + TAM density/phenotype + spatial CD8/TAM ratio + an exhaustion score, supplemented by on-treatment peripheral readouts (cytokines and/or ctDNA) to support adaptive combination selection.

To bridge the gap between multi−dimensional biomarkers and clinical practice, we propose the development of a composite “immune suppression score” that integrates key parameters from each level. For example, such a score could combine IDH mutation status (molecular), CD163^+^ TAM density and CD8^+^ T−cell abundance (cellular), the spatial CD8/TAM ratio at the tumor margin (spatial), and on−treatment changes in circulating cytokines or ctDNA (dynamic). This score could stratify patients into distinct immune microenvironment phenotypes and guide the selection of combination therapies. Several ongoing clinical trials are prospectively evaluating composite biomarker panels in glioma, and retrospective analyses of archived samples from completed immunotherapy trials (e.g., CheckMate−143) will be critical to validate their predictive value (73). Ultimately, transitioning from single markers to integrated signatures will be essential for precision immunotherapy in glioma.

## 6 Emerging immunotherapeutic strategies and their limitations in glioma

Based on the above understanding of immune-microenvironment subtypes, we now examine why current immunotherapies are broadly constrained across subtypes. In recent years, multiple immunotherapeutic approaches have entered clinical investigation for glioma, including immune checkpoint blockade, tumor vaccines, cellular immunotherapies, and oncolytic viruses (74). However, compared with other solid tumors, clinical benefit remains limited. Dissecting the shared causes of constrained efficacy helps reinterpret current therapeutic paths through the lens of the immune microenvironment and informs future optimization. Representative strategies and outcomes are summarized in **Table 2**.

**Table 2:** Representative clinical immunotherapy strategies and outcomes in glioma.

Strategy	Representative studies/phase	Primary population	Key outcomes	Shared reasons for limited benefit	Representative negative trials
ICIs (PD-1/PD-L1; CTLA-4)	Multiple phase II–III trials	Recurrent or newly diagnosed GBM	Limited overall survival improvement	Low tumor mutational burden, insufficient T-cell infiltration, myeloid-mediated immunosuppression	CheckMate-143 (nivolumab vs bevacizumab in recurrent GBM) (73); CheckMate-498 (nivolumab + RT vs RT + TMZ in newly diagnosed MGMT-unmethylated GBM) (75); NCT02017717 (ipilimumab + nivolumab in recurrent GBM) (76)
Tumor vaccines (peptide; dendritic cell [DC])	Phase I–II studies	Patients with specific antigen expression	Inducible immune responses, but clinical benefit unstable	High antigen heterogeneity, immune escape, insufficient durability of effector T cells	ACT IV trial (rindopepimut/EGFRvIII vaccine in newly diagnosed GBM) (77)
CAR-T therapy	Early-phase trials	EGFRvIII, IL13Rα2, etc.	Local or transient responses observed	Antigen loss, limited tumor infiltration, immunosuppressive microenvironment	NCT02209376 (EGFRvIII CAR-T in recurrent GBM) (78); NCT04077866 (IL13Rα2 CAR-T) (79)
Oncolytic virotherapy	Phase I–II studies	Recurrent GBM	Generally safe; efficacy variable	Limited viral spread, host antiviral clearance, insufficient immune activation	TARGET trial (DNX-2401 in recurrent GBM); NCT03152318 (PVSRIPO) (80)

Synthesis: The core challenge is not that “the immune system cannot be activated,” but that “activated immune effectors cannot sustain function within an immunosuppressive microenvironment,” indicating that single-agent approaches are unlikely to break immune tolerance in glioma.

Overall, the shared bottleneck is not inadequate immune activation per se, but the inability of immune effects to persist amid myeloid dominance, metabolism/hypoxia-driven suppression, and BBB-related constraints—highlighting the necessity of microenvironment remodeling and combination interventions.

### 6.1 Immune checkpoint inhibitors: immune activation does not equal relief of suppression

Inter-patient variability in ICI efficacy largely reflects differences in the overall immune-microenvironment state. Within the proposed integrative biomarker model, checkpoint blockade may yield limited benefit only in a minority of subtypes with relatively lower immunosuppression and some baseline T-cell infiltration and function (81). Yet in the overall glioma population, results have been disappointing. Multiple studies of PD-1/PD-L1 inhibitors show that monotherapy does not significantly improve overall survival. This reflects that immune suppression in glioma is not governed by a single checkpoint pathway but by coordinated multi-level mechanisms (9).

In glioma, T cells are often scarce and deeply exhausted; PD-1 blockade alone is frequently insufficient to restore effector function (82). Concurrently, immunosuppressive myeloid cells persist and secrete suppressive mediators, such that even “released” T cells cannot survive or function effectively within the TME (83). Therefore, ICI efficacy in glioma likely depends on combination strategies and optimized timing. Notably, although ICI monotherapy is limited in most immune-cold settings, neoadjuvant/early-stage administration and combinations with radiotherapy, anti-angiogenic therapy, or chemotherapy can improve the immune microenvironment and enhance clinical responses in subsets of patients (84, 85).

The disappointing results of phase III trials such as CheckMate−143 and CheckMate−498 underscore that PD−1 blockade alone is insufficient in unselected GBM populations (73, 75). These failures likely reflect the multifactorial nature of immune suppression, including low T−cell infiltration, profound T−cell exhaustion, and a dominant myeloid suppressive network that persists despite checkpoint blockade (47, 86).

### 6.2 Tumor vaccines: immune responses can be induced but are difficult to sustain

From the perspective of microenvironment subtyping, limited vaccine efficacy is not due to weak immune priming but to the inability of vaccine-induced immunity to persist within an immunosuppressive microenvironment. In myeloid-dominant and metabolically suppressive subtypes, enhancing antigen-specific responses alone often fails to translate into durable clinical benefit (87). Vaccine strategies in glioma include DC vaccines and personalized neoantigen vaccines (88). Some studies show measurable immune responses in subsets of patients, indicating that the immune system remains inducible (89).

However, immune activation often does not yield stable clinical benefit because the immunosuppressive microenvironment persists. Vaccine-induced T cells rapidly encounter inhibitory signals and metabolic constraints after entering the tumor, resulting in functional decline or apoptosis (45, 90). This highlights that enhancing recognition/activation without concurrently targeting suppressive factors is unlikely to achieve long-term efficacy (91).

While vaccine strategies have shown immunological activity in subsets of patients, none have yet demonstrated survival benefit in randomized trials. The failure of the Phase III ACT IV trial of the EGFRvIII vaccine (rindopepimut) in newly diagnosed GBM highlighted challenges such as antigen loss and the inability of vaccine−induced T cells to overcome a highly suppressive microenvironment (92).

### 6.3 Cellular immunotherapy: high precision but constrained by the microenvironment

Although cellular immunotherapies are highly targeted, their efficacy is similarly shaped by immune-microenvironment subtypes. In profoundly immunosuppressive, hypoxic, and nutrient-restricted environments, even successfully infused effector cells face major challenges in persistence and function (93). CAR-T and related approaches offer new possibilities (94). CAR-T therapies targeting antigens such as EGFRvIII and IL13Rα2 have shown acceptable safety and localized antitumor activity in early studies, supporting feasibility in glioma (95).

Nonetheless, antigen heterogeneity and immunosuppressive microenvironmental constraints remain major obstacles. Spatial and temporal heterogeneity can lead to antigen escape, while suppressive cytokines, metabolic restriction, and hypoxia directly impair CAR-T persistence and effector function (96). These issues imply that engineering improvements alone may be insufficient. Early clinical studies indicate that antigen-targeted cellular therapies can induce localized responses in some patients, but durability and eligible populations remain limited by immune suppression and tumor heterogeneity (97, 98).

Early−phase CAR−T trials have reported transient local responses, but durable benefit has been elusive. The lack of sustained efficacy is attributed not only to antigen heterogeneity but also to the rapid functional impairment of CAR−T cells upon encountering the immunosuppressive TME, as observed in trials targeting EGFRvIII and IL13Rα2 (78, 95).

### 6.4 Oncolytic viruses: a potential tool for microenvironment remodeling

Distinct from other strategies, a potential advantage of oncolytic viruses in glioma lies in their ability to directly impact the immune microenvironment. In immune-inert or myeloid-dominant suppressive subtypes, oncolytic viruses may promote immunogenic cell death and enhance innate immune activation, thereby creating more favorable conditions for subsequent immunotherapy (99, 100).

However, challenges remain, including delivery efficiency, antiviral immune clearance, and durability of efficacy (101). Balancing antitumor immune activation against excessive inflammation is a key issue for future studies. Overall, constrained efficacy across current immunotherapies is not due to isolated technical flaws but is tightly linked to multi-level microenvironmental blockade (102), further reinforcing the necessity of combination regimens and immune-microenvironment remodeling.

Despite promising preclinical data, oncolytic virotherapy has yet to achieve regulatory approval for GBM. The Phase III TARGET trial of DNX−2401 in recurrent GBM did not meet its primary endpoint, highlighting challenges related to viral delivery, antiviral immune clearance, and insufficient immune activation (101).

**Figure 2:**
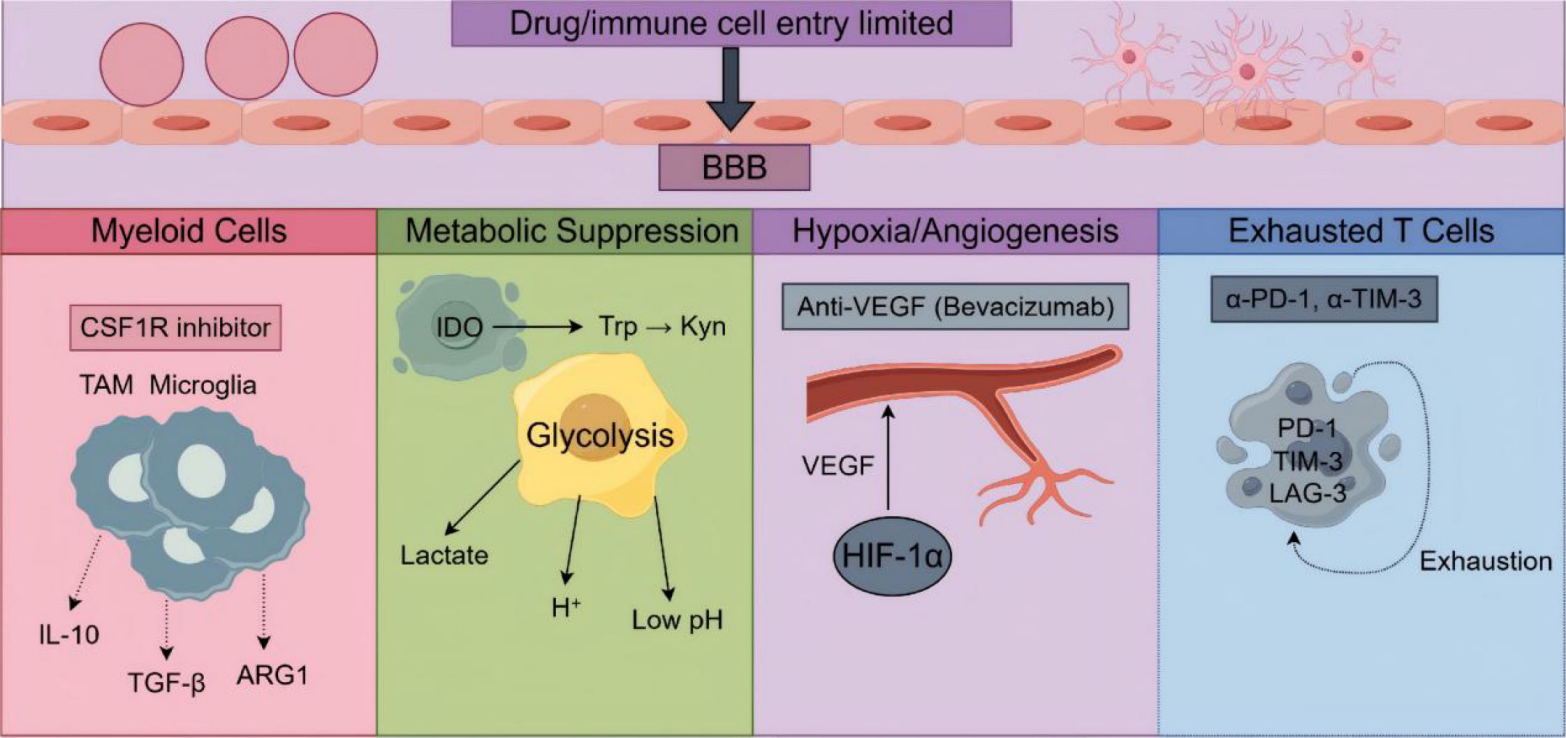
Remodeling the immunosuppressive tumor microenvironment in glioma. This schematic illustrates the key cellular components, molecular factors, and therapeutic targets within the immunosuppressive glioma microenvironment. The figure is organized into five functional compartments: the blood-brain barrier (BBB, top) restricts drug and immune cell entry into the brain parenchyma; the myeloid cell compartment (left) features tumor-associated macrophages (TAMs) and microglia, which are targeted by CSF1R inhibitors and produce immunosuppressive factors (IL-10, TGF-β, ARG1); the metabolic suppression compartment (left-center) shows glioma cells undergoing IDO-mediated tryptophan metabolism (Trp→Kyn) and glycolysis, generating lactate, H⁺, and low pH conditions; the hypoxia/angiogenesis compartment (right-center) depicts HIF-1α-driven VEGF expression and aberrant vascularization targeted by bevacizumab; and the exhausted T cell compartment (right) shows T cells expressing inhibitory receptors (PD-1, TIM-3, LAG-3) and an exhausted phenotype, with checkpoint inhibitors (α-PD-1, α-TIM-3) targeting these receptors.

A critical lesson from clinical trials of CSF1R inhibitors (e.g., PLX3397) is that depleting a single suppressive population is often insufficient to restore durable antitumor immunity. Despite substantial TAM reduction in preclinical models (33), clinical responses were limited (34). Several mechanisms may explain this failure: (1) compensatory recruitment of other myeloid cells, such as monocytic MDSCs or neutrophils (58); (2) upregulation of alternative immune checkpoints on remaining immune cells or tumor cells (86); (3) persistence of metabolic and hypoxic suppression even after TAM depletion (103); and (4) the possibility that TAM depletion also removes cells that could be reprogrammed toward an antitumor phenotype (54). These findings indicate that successful remodeling requires simultaneous targeting of multiple suppressive pathways rather than elimination of a single cell type (6, 104) (**Figure 2**).

### 6.5 Emerging immunotherapeutic strategies

Several novel approaches are entering clinical evaluation for glioma. Bispecific T−cell engagers (BiTEs) targeting EGFRvIII or IL13Rα2 are designed to redirect endogenous T cells to tumor cells (105). TAM−reprogramming strategies, such as CD40 agonists and PI3Kγ inhibitors, aim to convert immunosuppressive macrophages into immunostimulatory phenotypes (54). STING pathway agonists activate innate immunity and enhance T−cell priming, with preclinical efficacy in glioma models (61). Epigenetic modulators (e.g., HDAC inhibitors) can increase tumor immunogenicity (106). Finally, targeting the CSF immune interface via intrathecal delivery may overcome blood–brain barrier limitations (107). These strategies are at various stages of development and hold promise for future combination regimens.

## 7 Combination strategies: a key route to remodel the immunosuppressive glioma microenvironment

Given that the immunosuppressive glioma microenvironment is maintained by multiple cell types, molecular signals, and metabolic pathways, single-agent approaches rarely achieve durable benefit. A growing consensus is that combination therapy is not a simple additive stacking of modalities; rather, it aims to remodel the microenvironment through multi-level synergistic interventions, enabling effective immune responses (108).

An emerging concept in glioma immunotherapy is that the timing of intervention critically shapes outcomes. The immunosuppressive microenvironment becomes increasingly entrenched as the tumor progresses and in response to treatment. Therefore, neoadjuvant (pre−surgical) or early−adjuvant administration may exploit a window of lower tumor burden and a less rigidly established suppressive network, potentially yielding more durable immune responses (109, 110). In contrast, immunotherapy given at recurrence often faces a microenvironment that has been extensively remodeled by prior therapies and is dominated by profound immunosuppression (47). Consequently, when designing combination regimens, the sequence and timing of each modality must be considered as a core variable, not an afterthought.

### 7.1 Immunotherapy plus radiotherapy: enhancing immunogenicity and antigen presentation

Radiotherapy is a cornerstone of standard glioma treatment, and its immunomodulatory effects have attracted increasing attention. Radiotherapy can directly kill tumor cells and induce immunogenic cell death, promoting tumor-antigen release and enhancing antigen presentation (111). These effects provide potential synergy with ICIs and other immunotherapies (112).

In glioma, combining radiotherapy with immunotherapy may partially overcome low immunogenicity. However, radiotherapy can also induce immunosuppressive signals and recruit suppressive cells, making dose, sequencing, and regimen design critical (113, 114). A key challenge is enhancing immune activation without exacerbating immune suppression.

The sequence of radiotherapy and immunotherapy is critical. Preclinical studies suggest that delivering radiotherapy before immune checkpoint blockade may optimize antigen release and presentation, while concurrent administration could risk depleting lymphocytes (111, 114). Neoadjuvant radiotherapy followed by immunotherapy is being explored in early−phase trials (e.g., NCT03576612) (115).

### 7.2 Immunotherapy plus anti-angiogenic therapy: improving immune-cell entry and function

Abnormal tumor vasculature not only supports glioma growth but also limits effective immune-cell entry into tumor tissue (116). Anti-angiogenic therapy may promote “vascular normalization,” improving perfusion and oxygenation, relieving hypoxia-associated immune suppression, and facilitating immune infiltration (117).

Combining immunotherapy with anti-angiogenic therapy may improve both physical access and functional states of immune cells. Some studies suggest improved efficacy of ICIs in glioma, but clinical benefit requires validation in larger cohorts (118). From a microenvironmental perspective, this strategy may be particularly suitable for patients with pronounced vascular abnormalities, imaging evidence of poor perfusion, or high hypoxia burden (119). In such contexts, vascular normalization may create more permissive conditions for immunotherapy (120).

Anti−angiogenic agents induce a transient “normalization window” of improved tumor perfusion and reduced hypoxia (121). Combining immunotherapy during this window may enhance immune cell infiltration and function. Timing is therefore crucial; administering immunotherapy too early or too late may miss this therapeutic opportunity (122).

### 7.3 Myeloid-targeted combinations: disrupting the core immunosuppressive hub

Given the central role of myeloid cells in glioma immune suppression, targeting TAMs and related signaling pathways is considered a critical entry point for remodeling (123). Strategies that inhibit myeloid recruitment, block suppressive functions, or reprogram myeloid cells toward immune-activating phenotypes may create a more favorable environment for T-cell–mediated immunity (124).

Combining myeloid-targeting approaches with ICIs or cellular therapies is theoretically advantageous (125). However, because myeloid populations are heterogeneous and essential for physiological functions, achieving precise intervention without systemic immune dysregulation remains challenging. Early clinical exploration suggests measurable signals in “altering the microenvironment to facilitate ICIs,” but demonstrating stable, reproducible survival benefit requires biomarker-stratified prospective trials (126). Such combinations may be especially beneficial in suppressive subtypes with high TAM infiltration and low CD8^+^ T cell/TAM ratios, offering a path to dismantle the suppressive hub and restore adaptive immunity (104).

Myeloid−targeted therapies may be most effective when initiated before the establishment of a dense immunosuppressive network (127). Neoadjuvant CSF1R inhibition, for example, could deplete TAMs prior to definitive surgery, creating a more permissive environment for subsequent T−cell−based therapies (33).

### 7.4 Immunotherapy plus metabolic intervention: relieving functional suppression

Metabolic reprogramming is a core mechanism sustaining glioma immune suppression. Interventions targeting lactate metabolism, the tryptophan–kynurenine (Kyn) axis, and hypoxia-adaptation pathways can improve immune-cell survival and effector states within the TME. Combining metabolic modulation with immunotherapy may relieve functional suppression and support more durable antitumor immunity (50, 128).

Overall, multimodal combination therapy represents a key future direction. The goal is not to maximize the number of combined modalities, but to rationally target pivotal nodes of immune suppression to achieve true immune remodeling.

**Figure 3:**
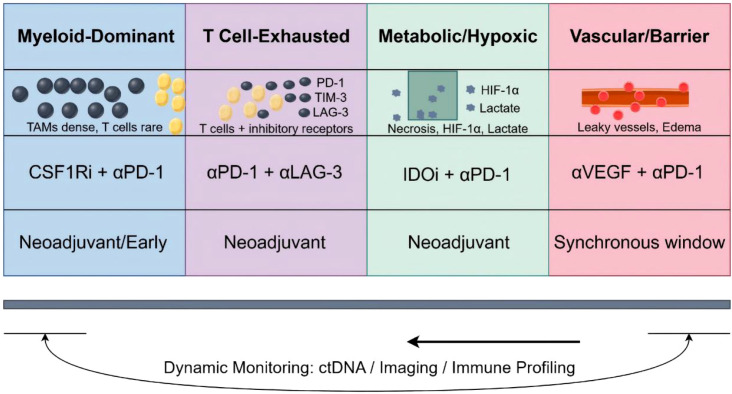
Microenvironment-informed decision framework. This schematic presents a decision framework for selecting combination immunotherapies based on glioma immune microenvironment subtypes. Patients are stratified into four subtypes according to dominant microenvironmental features: myeloid-dominant (dense TAM infiltration with sparse T cells), T cell-exhausted (T cells present but expressing inhibitory receptors PD-1/TIM-3/LAG-3), metabolic/hypoxic (necrosis with HIF-1α and lactate accumulation), and vascular/barrier (leaky vessels and edema). For each subtype, the preferred combination strategy and optimal treatment timing are indicated. Dynamic monitoring using ctDNA, imaging, and immune profiling enables adaptive therapy adjustment throughout treatment, with results feeding back to guide re-stratification and treatment modification.

Metabolic interventions (e.g., IDO inhibition, lactate targeting) may be most beneficial when combined with immunotherapy early in the treatment course, before metabolic reprogramming becomes fully entrenched and drives profound immune suppression (31, 103) (**Figure 3**).

### 7.5 Suggested (not prescriptive) algorithm for microenvironment-informed immunotherapy

A suggested (not prescriptive) framework for microenvironment-informed immunotherapy is as follows: First, perform baseline profiling by integrating molecular, cellular, spatial, and dynamic immune readouts; Second, categorize the microenvironment by identifying dominant barriers, including myeloid-dominant suppression, T-cell exhaustion, metabolic or hypoxic suppression, and/or therapeutic delivery constraints; Third, match strategies accordingly (examples), prioritizing myeloid-targeting combinations for myeloid-dominant tumors, reinforcing immune checkpoint blockade and/or immune priming and optimizing treatment timing for exhaustion-dominant tumors, considering metabolic or vascular normalization strategies for hypoxic or metabolically suppressive microenvironments, and considering local delivery or oncolytic approaches for delivery-limited settings; and finally, conduct on-treatment reassessment by tracking core composite indicators (e.g., spatial CD8/TAM ratio, T-cell exhaustion profiles, and peripheral immune dynamics) and adapting therapeutic combinations as needed. This framework is provided for conceptual guidance only and is not prescriptive; clinical application should be aligned with trial availability, patient-specific factors, and safety considerations.

## 8 Challenges and future perspectives

Despite deepening understanding of the immunosuppressive glioma microenvironment and active exploration of immunotherapies and combinations, clinical translation faces major challenges. These challenges reflect not a single technical limitation but the combined effects of tumor biological complexity, delicate immune regulation, and trial-design constraints.

First, pronounced spatial and temporal heterogeneity remains a major barrier. Immune-microenvironment features can differ significantly across patients, across tumor regions, and across disease stages within the same patient (69). This heterogeneity complicates universal applicability of any single strategy and increases difficulty in predicting response and optimizing regimens.

Second, the lack of reliable predictive biomarkers severely constrains precision use of immunotherapy. Most candidate immune biomarkers have not been sufficiently validated in glioma, and single molecular or cellular indicators often fail to represent global microenvironment states (129). Future studies should rely more on multi-omics integration, dynamic monitoring, and spatial analyses to build composite evaluation systems that reflect degree of immune suppression and treatment sensitivity.

Third, immune-related toxicity and neurological safety are particularly critical in glioma. The CNS is highly sensitive to inflammation, and balancing antitumor immune activation against severe neurotoxicity is a key consideration. This imposes stringent requirements on dosing, sequencing, and patient selection for combination regimens (130, 131).

In addition, complexity in clinical-trial design affects evaluation of glioma immunotherapies. Patient histories are heterogeneous, disease progression is rapid, and endpoints and response criteria remain debated. Timing and sequencing of immunotherapy are emerging as key issues. Neoadjuvant immunotherapy, adjuvant settings, and optimization relative to standard chemoradiotherapy may produce very different microenvironmental effects (132). Increasing evidence suggests that immune intervention at low tumor burden—before immune suppression becomes “locked”—may more readily induce strong and durable immunity; once high-burden, deeply suppressive states dominate, more complex combinations and “de-suppression” strategies are required (133, 134). In rapidly progressive, strongly immunosuppressive glioma, balancing intensity, CNS safety, and the immune-activation window, while incorporating microenvironmental changes as biological endpoints, remains an urgent unresolved challenge.

Looking forward, glioma immunotherapy is likely to shift from “trying new therapies” to “precision remodeling of the immune microenvironment.” By identifying key nodes of the suppressive network, rationally integrating modalities, and applying strategies at the right time to the right patients, immunotherapy may yield more substantive clinical impact.

## 9 Conclusions

A refractory immunosuppressive microenvironment is a fundamental reason why immunotherapy has repeatedly fallen short in glioma. A large body of basic and clinical evidence indicates that simply boosting immune activation or relying on single-agent immunotherapy is insufficient to overcome immune suppression maintained by myeloid dominance, multi-level signaling control, and metabolic reprogramming.

Guided by the core concept of “immune-microenvironment remodeling,” dissecting key suppressive mechanisms, identifying clinically meaningful predictive biomarkers, and developing combination-oriented immune interventions are essential paths forward. Future immunotherapy should not be viewed as an isolated modality, but as an integral component of an integrated treatment system that works synergistically with standard-of-care therapies.

Overall, remodeling—rather than merely activating—immune responses is likely a prerequisite for breakthroughs in glioma immunotherapy. With advances in immunologic technologies and optimized clinical research, precise intervention of the immunosuppressive microenvironment may offer glioma patients more durable and effective therapeutic benefits.
